# Association between the participation of the plastic surgery department and qualitative prognoses in severe trauma patients: A retrospective observational study

**DOI:** 10.1097/MD.0000000000032387

**Published:** 2022-12-23

**Authors:** Nam Kyu Lim, Sungyeon Kim, Jae Hee Yoon, Kyung-Hwa Choi

**Affiliations:** a Department of Plastic and Reconstructive Surgery, Dankook University Hospital, Cheonan, Chungnam, Republic of Korea; b Department of Plastic and Reconstructive Surgery, Dankook University College of Medicine, Cheonan, Chungnam, Republic of Korea; c Department of Preventive Medicine, Dankook University College of Medicine, Cheonan, Chungnam, Republic of Korea.

**Keywords:** plastic surgery, prognosis, trauma centers

## Abstract

Catastrophic incidents would necessitate the intervention of multiple specializations with plastic surgery (PS) as an indispensable area of expertise. In view of PS, prognostic assessment of trauma patients should be focused on the qualitative value rather than mortality because plastic surgeons rarely handled patients’ vital signs in actual. Thus, we explored the association between the involvement of the PS department and qualitative prognoses for severe trauma patients. From November 2014 to December 2019, we enrolled total 529 trauma patients with an injury severity score (ISS) over 15 points. We set the prognostic factors that the rate of admission in intensive care unit (ICU), total or ICU duration of hospitalization, post-discharge progress and disability diagnosis which were regarded as qualitative prognoses. The analysis was performed with logistic regression analysis or regression analysis adjusted for age, sex, past medical history, cause of trauma, and frequency of operation. Among total of 529 patients, 290 patients in PS group and 239 patients in non-PS group were analyzed. In both groups, the under-65-year ages and male patients were significantly predominant. The rate of going home showed 2.082 times higher in PS group than non-PS group after adjusting for covariates, while there was no significant difference in diagnosis of disability. Meanwhile, overall prognoses were highly correlated with either higher ISS or lower Glasgow Coma Scale (GCS). In conclusion, higher severity generally affected to the severe trauma patient’s prognoses, and the PS treatment only contributes to discharge disposition to home.

## 1. Introduction

In the recent decades, disasters of massive scale, such as the 1995 Oklahoma City bombing, the attack on the World Trade Center in 2001, and the 2005 London Underground bombing, have raised disaster awareness not only across health professions but also among the public in general. Such experiences have thrown light on the crucial role of surgical specialists, most particularly that of plastic surgeons.^[1]^ Given the variety of injuries found in victims of such disasters, many patients require plastic surgery (PS); a case in point is the 1999 Turkey earthquake, where more than 13% of the hospitalized needed care from plastic surgeons. In general, patients who are treated for acute trauma should subsequently be examined and managed by plastic surgeons, who will close their wounds with grafts, flaps, or by transferring adjacent tissue.^[2]^

Even an isolated case of trauma calls for the collaboration of a team led by trauma surgeons and emergency medicine physicians, who work with specialists from the disciplines of neurosurgery, orthopedists, critical care, PS, and many others. As general trauma requires a multidisciplinary response, catastrophic incidents with large numbers of casualties would also necessitate the intervention of a team of medical professionals from multiple specializations, with PS as an indispensable area of expertise.^[1]^

Plastic surgeons are experienced and knowledgeable in burns and trauma of soft tissue, face and extremities, the most common injuries encountered in natural disasters. Though not always life-threatening, these wounds, when not properly managed, may leave lasting functional complications.^[2]^ This underscores the role of plastic surgeons in improving the qualitative prognoses for trauma patients.

We note that existing literature on trauma mostly deals with initial assessments, with few studies discussing qualitative prognoses. This study aims to explore the association between the involvement of the PS department and qualitative prognoses for severe trauma patients, and will establish the contribution of plastic surgeons in treatment of major trauma.

## 2. Methods

### 2.1. Study subjects

**Figure 1.**
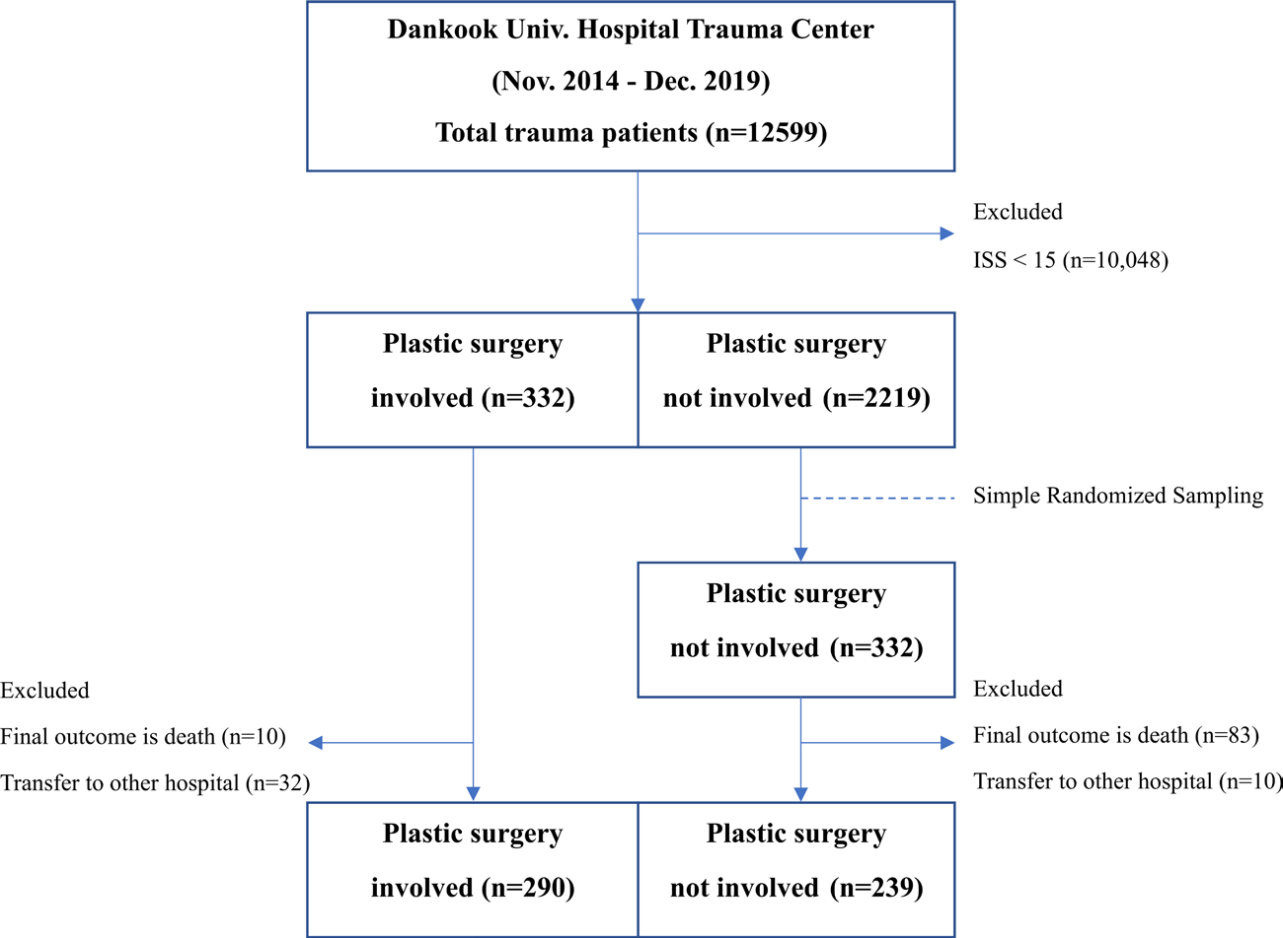
Sampling algorithm of the number of participants in the study.

We designed a retrospective study to review the data for severe trauma patients (Injury Severity Score [ISS] > 15 points) in the Trauma Registry System of our hospital from November 2014 to December 2019. Following institutional guidelines, this study was approved by the Institutional Review Board of Dankook University Hospital (IRB No. 2021-04-021). Of the 2551 total patients during study period, 332 were treated by specialists from the PS department. To match the number of samples in each group, we extracted 332 patients among the 2219 patients who were not treated by the PS department (non-PS) using a simple randomized sampling method. Forty-two patients in the PS group and 93 in the non-PS group were excluded because they died or were transferred during hospitalization. After exclusion, a total of 290 patients in the PS group and 239 patients in the non-PS group were enrolled in the study (Fig. 1). The IRB waived all patients’ consent due to the retrospective nature of the study.

### 2.2. Severity score

In trauma patients, severity is usually evaluated anatomically, physiologically, and using a combination of the two. Among the available assessment tools, we used the ISS as an anatomical indicator and the Glasgow Coma Scale (GCS) as a physiological indicator. ISS is calculated by adding the squares of the top 3 categories of the Abbreviated Injury Scale (AIS), and the score ranges from 0 to 75. Generally, the score is divided into 6 groups (1–8, minor; 9–15, moderate; 16–24, serious; 25–49, severe; 50–74, critical; and 75, untreatable), with higher scores indicating a higher risk of mortality. Patients with a score over 15 points are usually classified as having severe trauma. In contrast, for the GCS, a lower score indicates a worse prognosis, with a score of 13 to 15 points indicating a mild condition, 9 to 12 points indicating a moderate condition, and a score of 3 to 8 points indicating a severe condition. In our study, in which we analyzed a group of patients with an ISS over 15 points, the patients were divided into 2 ISS groups (based on a score of 25 points) and 2 GCS groups (based on a score of 13 points). In addition, of the 6 AIS categories (head and neck, face, chest, abdomen, extremity, and external), we further analyzed those with facial injuries.

### 2.3. Prognoses

We set the prognostic factors such as the rate of admission in the intensive care unit (ICU), total length of stay (LOS), ICU LOS, post-discharge disposition (home or convalescent hospital), and disability diagnosis. Among them, post-discharge disposition and disability diagnosis were regarded as qualitative prognostic indicators. Disability diagnosis was determined based on the disability certificate that was prepared either during admission or after discharge, from any department.

### 2.4. Covariates

Additional data on all trauma patients were retrieved from the Trauma Registry System of the study institution, which included age, sex, past medical history, cause of trauma, and frequency of operations. Age was divided into 2 groups according to the age of 65 years. Past medical history included hypertension, diabetes mellitus, tuberculosis, and hepatitis. The causes of trauma included traffic accidents, falls, occupational accidents, and assaults. The group classified as “etc.” included events such as slip down, suicide attempts, loss of consciousness, and unknown. The frequency of operations was divided into 3 groups (0, 1, and ≥2).

### 2.5. Statistical analysis

A chi-square test was performed to establish whether PS was associated with the covariates and severity factors. Chi-square tests, *t* tests, and ANOVAs were performed to establish whether covariates were associated with prognosis and severity factors. Logistic regression was conducted to compare the prognosis for PS participation and severity factors (individually or grouped), after adjusting for age, sex, past medical history, cause of trauma, and frequency of operations. All statistical analyses were performed using IBM SPSS software version 23 (IBM Corp., Armonk, NY). Statistical significance was set at *P* < .05.

## 3. Results

### 3.1. Demographic comparison between groups (PS vs Non-PS, Table 1)

Of the 529 patients analyzed, 290 and 239 patients were included in the PS and Non-PS groups, respectively. The under-65-year ages (77.6% vs 66.1%) and male (82.4% vs 72.0%) were significantly predominant in both groups, and a significant difference was found between the groups (*P* < .005). Most of the patients in both groups had no past medical history (78.3% vs 64.4%, *P* < .001). The causes of trauma were also similar in both groups, with traffic accidents being the most common cause (64.9% vs 54.4%), followed by falls (17.2% vs 17.6%). However, the frequency of operations was not significantly associated with PS involvement (*P* = .299).

**Table 1:** Characteristics of severe trauma patients by plastic surgery (PS) treatment.

Variable	Non-PS (n = 239)	PS (n = 290)	*P* value
	N (%)	N (%)	
Age (yrs)			**.005**
<65	158 (66.1)	225 (77.6)	
≥65	81 (33.9)	65 (22.4)	
Sex			**.005**
Male	172 (72.0)	239 (82.4)	
Female	67 (28.0)	51 (17.6)	
Past medical history*			**<.001**
Yes	85 (35.6)	63 (21.7)	
No	154 (64.4)	227 (78.3)	
Cause of trauma			**.001**
Traffic accident	130 (54.4)	188 (64.8)	
Falls	42 (17.6)	50 (17.2)	
Occupational	25 (8.6)	25 (8.6)	
Assault	2 (0.8)	7 (2.4)	
Etc.†	44 (18.4)	20 (6.9)	
Frequency of operation			.299
0	69 (28.9)	67 (23.1)	
1	71 (29.7)	89 (30.7)	
≥2	99 (41.4)	134 (46.2)	
GCS			.391
≥13	193 (80.8)	238 (82.1)	
<13	46 (19.2)	52 (17.9)	
ISS			**<.001**
<25	136 (56.9)	218 (75.2)	
≥25	103 (43.1)	72 (24.8)	
AIS (face)			**<.001**
Yes	42 (17.6)	228 (84.4)	
No	197 (82.4)	62 (21.4)	

n, number; Data are presented as n (%).

* Including hypertension, diabetes mellitus, tuberculosis, and hepatitis.

† Including slip down, suicide attempts, loss of consciousness, unknown.

AIS = abbreviated injury score, GCS = Glasgow coma scale, ISS = injury severity score.

*P* value estimated using chi-square test

Regarding the severity indicators between the 2 groups, while the GCS category showed no significant differences (*P* = .391), ISS categories were significantly different between the groups. In particular, the subgroup of patients with an ISS < 25 was dominant in the PS group compared with the non-PS group (75.2% vs 56.9%, *P* < .001). In addition, the patients in the PS group were more likely to have face involvement on the AIS than those in the non-PS group (84.4% vs 17.6%, *P* < .001).

### 3.2. Distribution of trauma severity and covariates (Table 2)

Neither the ISS nor GCS scores were significantly different according to age (*P* = .918 and *P* = .999, respectively) or sex (*P* = .437 and *P* = .999, respectively). In contrast, the involvement of the face on the AIS was significantly associated with sex (*P* = .037). In the analysis according to the cause of trauma, both the ISS and facial injury on the AIS were significantly different (*P* = .013 and *P* = .003, respectively).

**Table 2:** Distribution of severity by general characteristics among severe trauma patients.

Variable	All (n)	ISS		GCS		AIS (face)	
		<25	≥25	≥13	<13	Yes	No
	N	N (%)	N (%)	N (%)	N (%)	N (%)	N (%)
All (n)	529	354	175	431	98	270	259
Age (yrs)							
<65	383	257(72.6)	126(72.0)	312(72.4)	71(72.4)	201(74.4)	182(70.3)
≥65	146	97(27.4)	49(28.0)	119(27.6)	27(27.6)	69(25.6)	77(29.7)
*P* value		.918		.999		.287	
Sex							
Male	411	271(76.6)	140(80.0)	335(77.7)	76(77.6)	220(81.5)	191(73.7)
Female	118	83(23.4)	35(20.0)	96(22.3)	22(22.4)	50(18.5)	68(26.3)
*P* value		.437		.999		**.037**	
Past medical history*							
Yes	148	100(28.2)	48(27.4)	124(28.8)	24(24.5)	66(24.4)	82(31.7)
No	381	254(71.8)	127(72.6)	307(71.2)	74(75.5)	204(75.6)	177(68.3)
*P* value		.918		.455		.067	
Cause of trauma							
TA	318	224(63.3)	94(53.7)	261(60.5)	57(58.1)	176(65.2)	142(54.8)
Falls	92	58(16.4)	34(19.4)	74(17.2)	18(18.4)	44(16.3)	48(18.5)
Occupational	46	29(8.2)	17(9.7)	42(9.7)	4(4.1)	20(7.4)	26(10.0)
Assault	9	9(2.5)	0(0.0)	8(1.9)	1(1.0)	8(3.0)	1(0.4)
Etc.†	64	34(9.6)	30(17.2)	46(10.7)	18(18.4)	22(8.1)	42(16.2)
*P* value		**.013**		.120		**.003**	
Frequency of operation (n)							
0	136	97(27.4)	39(22.3)	112(26.0)	24(24.5)	67(24.8)	69(26.6)
1	160	115(32.5)	45(25.7)	137(31.8)	23(23.5)	79(29.3)	81(31.3)
≥2	233	142(40.1)	91(52.0)	182(42.2)	51(52.0)	124(45.9)	109(42.1)
*P* value		**.035**		.162		.673	

n = number; Data are presented as n (%).

* Including hypertension, diabetes mellitus, tuberculosis, and hepatitis

† Including slip down, self-injury, loss of consciousness, unknown.

AIS = abbreviated injury score, GCS = Glasgow coma scale, ICU = intensive care unit, ISS = injury severity score, LOS = length of stay, TA = traffic accident.

*P* value estimated using chi-square test, *t* test, or ANOVA.

### 3.3. Distribution of prognosis and covariates (Table 3)

The mean duration of hospitalization (total LOS) was 34.2 ± 32.2 days for all 529 patients, 431 of which (81.5%) entered the ICU, with an average ICU LOS of 7.8 ± 13.2 days. These quantitative outcomes were generally not significantly associated with the covariates (e.g., age, past medical history, and cause of trauma); however, there were significant differences in the rate of ICU admission (*P* = .004) and ICU LOS (*P* = .001) according to sex.

**Table 3:** Distribution of prognosis by general characteristics among severe trauma patients.

Variable	All (n)	Total LOS	ICU LOS	ICU admission		Discharge disposition (Home)		Disability diagnosis	
				Yes	No	Yes	No	Yes	No
	N	Mean ± SD	Mean ± SD	N (%)	N (%)	N (%)	N (%)	N (%)	N (%)
All (n)	529	34.2 ± 32.2	7.8 ± 13.2	431	98	246	283	102	427
Age (yrs)									
<65	383	35.2 ± 33.0	7.2 ± 12.1	309(71.7)	74(75.5)	193(78.5)	190(67.1)	77(75.5)	306(71.7)
≥65	146	31.7 ± 29.6	9.2 ± 15.5	122(28.3)	24(24.5)	53(21.5)	93(32.9)	25(24.5)	121(28.3)
*P* value		.259	.175	.531		**.005**		.462	
Sex									
Male	411	35.6 ± 33.5	8.5 ± 14.1	346(80.3)	65(66.3)	187(76.0)	224(79.2)	84(82.4)	327(76.6)
Female	118	29.7 ± 26.6	5.0 ± 8.8	85(19.7)	33(33.7)	59(24.0)	59(20.8)	18(17.6)	100(23.4)
*P* value		.080	**.001**	**.004**		.404		.235	
Past medical history*									
Yes	148	32.5 ± 28.8	8.1 ± 12.7	118(27.4)	30(30.6)	53(21.5)	95(33.6)	30(29.4)	118(27.6)
No	381	34.9 ± 33.4	7.6 ± 13.4	313(72.6)	68(69.4)	193(78.5)	188(66.4)	72(70.6)	309(72.4)
*P* value		.447	.694	.534		**.003**		.714	
Cause of trauma									
TA	318	34.7 ± 33.0	7.8 ± 13.8	255(59.2)	63(64.3)	143(58.1)	175(61.8)	63(61.8)	255(59.7)
Fall down	92	36.0 ± 31.8	7.8 ± 11.1	75(17.4)	17(17.3)	43(17.5)	49(17.3)	19(18.6)	73(17.1)
Occupational	46	36.3 ± 36.4	6.2 ± 11.4	40 (9.3)	6 (6.1)	21 (8.5)	25 (8.8)	11(10.8)	35 (8.2)
Assault	9	27.3 ± 21.3	8.9 ± 19.7	8(1.8)	1(1.0)	7(2.9)	2(0.7)	1(1.0)	8(1.9)
Etc.†	64	29.0 ± 26.0	8.7 ± 13.3	53(12.3)	11(11.2)	32(13.0)	32(11.3)	8(11.3)	56(13.1)
*P* value		.618	.907	.797		.384		.545	
Frequency of operation (n)									
0	136	14.3 ± 12.0	4.2 ± 8.3	111(25.7)	25(25.5)	67(27.2)	69(24.4)	11(10.8)	125(29.3)
1	160	25.8 ± 18.0	4.6 ± 6.9	124(28.8)	36(36.7)	84(34.1)	76(26.9)	28(27.4)	132(30.9)
≥2	233	51.6 ± 38.3	12.0 ± 17.0	196(45.5)	37(37.8)	95(38.6)	138(48.8)	63(61.8)	170(39.8)
*P* value		**<.001**	**<.001**	.252		.055		**<.001**	

n = number; Data are presented as n (%) and mean ± standard deviation.

* Including hypertension, diabetes mellitus, tuberculosis, and hepatitis.

† Including slip down, self-injury, loss of consciousness, unknown.

AIS = abbreviated injury score, GCS = Glasgow coma scale, ICU = intensive care unit, ISS = injury severity score, LOS = length of stay, TA = traffic accident.

*P* value estimated using chi-square test, *t* test, or ANOVA.

In the qualitative analysis of prognosis, which included discharge disposition (home) and disability diagnosis, the rate of discharge to home was significantly correlated with the under-65-year age group (*P* = .005) and the group with no past medical history (*P* = .003). However, disability diagnosis was only significantly correlated with the frequency of operation (*P* < .001).

### 3.4. Multivariate regression analysis of prognosis (Table 4)

For the multivariate logistic regression, 3 models were analyzed to examine the relationship between PS department participation, severity indicators, and prognostic factors.

**Table 4:** Multivariate regression analysis for prognosis by severity and plastic surgery (PS) treatment among severe trauma patients.

		Total LOS				ICU LOS				ICU admission (Yes)				Discharge disposition (Home)				Disability (Yes)			
		N	β	SE	*P* value	N	β	SE	*P* value	N	OR	95% CI		N	OR	95% CI		N	OR	95% CI	
Non-PS		239	Ref			239	Ref			203	Ref			86	Ref			41	Ref		
PS		290	−0.320	2.540	.192	290	−0.238	1.107	.829	228	0.737	0.445	1.221	160	**2.082**	**1.421**	**3.050**	61	1.298	0.807	2.087
ISS	<25	354	ref			354	ref			261	ref			183	ref			60	ref		
	≥25	175	**10.827**	**2.641**	**<.001**	175	**7.871**	**1.151**	**<.001**	170	**11.848**	**4.658**	**30.137**	63	**0.602**	**0.404**	**0.895**	42	1.546	0.961	2.484
Non-PS		239	ref			239	ref			203	ref			86	ref			41	ref		
PS		290	−5.836	3.147	.064	290	−2.383	1.409	.091	228	0.659	0.368	1.180	160	1.462	0.922	2.317	61	1.436	0.816	2.527
AIS (face)	No	259	1			259	1			218	ref			92	ref			51	ref		
	Yes	270	0.964	3.091	.755	270	1.223	1.383	.377	213	0.801	0.456	1.406	154	**2.091**	**1.328**	**3.291**	51	0.728	0.419	1.262
Non-PS		239	ref			239	ref			203	ref			86	ref			41	ref		
PS		290	−**5.186**	**2.512**	**.039**	290	−1.577	1.081	.145	228	**0.584**	**0.358**	**0.951**	160	**2.272**	**1.557**	**3.315**	61	1.208	0.758	1.925
GCS	≥13	431	ref			431	ref			336	ref			212	ref			76	ref		
	<13	98	**9.910**	**3.140**	**.002**	98	**9.878**	**1.351**	**<.001**	95	**9.011**	**2.772**	**29.293**	34	**0.526**	**0.325**	**0.850**	26	**1.759**	**1.029**	**3.007**

*β* = regression coefficient, AIS = abbreviated injury score, CI = confidence interval, GCS = Glasgow coma scale, ICU = intensive care unit, ISS = injury severity score, LOS = length of stay, N = number, OR = odds ratio, SE = standard error.

β, SE, and *P* value estimated using regression analysis adjusted for age, sex, past medical history, cause of trauma, frequency of operation.

OR and 95% CI estimated using logistic regression analysis adjusted for age, sex, past medical history, cause of trauma, frequency of operation.

The first model was performed to assess the relationship between the participation of the PS department and the ISS. For the quantitative prognostic indicators (total LOS, ICU LOS, and the rate of ICU admission), a significant correlation was found according to the ISS but not between the PS and non-PS groups. The group with an ISS ≥ 25 had a total LOS that was 10.827 days longer (*P* < .001), an ICU LOS that was 7.872 days longer (*P* < .001), and a rate of ICU admission that was 11.848 times (95% CI = 4.658–30.137) that of the group with an ISS < 25. However, for the qualitative prognostic indicators, the rate of discharge to home in the PS group was 2.082 times that in the non-PS group (95% CI = 1.421–3.050), and the rate in the ISS ≥ 25 group was 0.602 times that in the ISS < 25 group (95% CI = 0.404–0.895). However, there were no significant differences according to disability diagnosis.

The second model was conducted to assess the relationship between the participation of the PS department and facial injury on the AIS. Unlike the first model, there were no significant differences between the groups except for the rate of discharge to home (OR = 2.091, 95% CI = 1.328–3.291).

The last model was performed to assess whether the participation of the PS department and the GCS score were correlated. Both were significantly correlated with the total LOS (β = −5.186, *P* = .039 for the PS group and β = 9.910, *P* = .002 for the GCS < 13 group) and the rate of discharge to home (OR = 2.272, 95% CI = 1.557–3.315 for the PS group and OR = 0.526, 95% CI = 0.325–0.850 for the GCS < 13 group). In contrast, only the group with a GCS score < 13 was significantly correlated with a disability diagnosis (OR = 1.759, 95% CI = 1.029–3.007).

### 3.5. Joint effect of PS involvement and trauma severity on prognosis (Table 5)

To examine the joint effect of the involvement of the PS department and the severity indicators on prognosis, we categorized the patients into 4 subgroups, 2 for each variate. For this analysis, there were also 3 models.

**Table 5:** Joint effect between plastic surgery (PS) treatment and severity on prognosis among severe trauma patients.

		N	Total LOS			ICU LOS			ICU admission (Yes)			Discharge disposition (Home)			Disability (Yes)		
			β	SE	*P* value	β	SE	*P* value	OR	95% CI		OR	95% CI		OR	95% CI	
Non-PS	ISS ≥ 25	103	ref			ref			ref			ref			ref		
	ISS < 25	136	−**8.780**	**3.684**	**.018**	−**5.246**	**1.598**	**.001**	**0.095**	**0.028**	**0.326**	1.391	0.791	2.447	0.657	0.325	1.332
PS	ISS ≥ 25	72	−0.534	4.322	.902	3.335	1.875	.076	0.944	0.151	5.920	1.650	0.858	3.174	1.320	0.636	2.741
	ISS<25	218	−**13.510**	**3.403**	**<.001**	−**7.292**	**1.476**	**<.001**	**0.069**	**0.020**	**0.231**	**3.243**	**1.919**	**5.480**	0.843	0.456	1.558
Non-PS	AIS (No)	197	ref			ref			ref			ref			ref		
	AIS (Yes)	42	−3.724	4.714	.430	0.713	2.155	.741	0.907	0.358	2.298	1.832	0.901	3.724	0.529	0.189	1.481
PS	AIS (No)	62	−**8.416**	**4.086**	**.040**	−2.761	1.868	.140	0.717	0.330	1.561	1.325	0.716	2.451	1.222	0.598	2.494
	AIS (Yes)	228	−2.935	2.822	.299	−1.177	1.263	.352	**0.533**	**0.313**	**0.908**	**3.035**	**1.985**	**4.640**	1.033	0.619	1.725
Non-PS	GCS<13	46	ref			ref			ref			ref			ref		
	GCS ≥ 13	193	−4.146	4.604	.368	−**7.445**	**1.982**	**<.001**	0.308	0.088	1.076	**2.144**	**1.001**	**4.593**	0.840	0.358	1.971
PS	GCS < 13	52	3.604	5.722	.529	2.134	2.463	.387	**9.845**	**0.215**	**0.458**	**2.694**	**1.087**	**6.679**	2.030	0.765	5.390
	GCS ≥ 13	238	−**11.264**	**4.583**	**.014**	−**9.837**	**1.973**	**<.001**	**0.162**	**0.047**	**0.562**	**4.714**	**2.203**	**10.085**	0.874	0.379	2.016

*β* = regression coefficient, AIS = abbreviated injury score, CI = confidence interval, GCS = Glasgow coma scale, ICU = intensive care unit, ISS = injury severity score, LOS = length of stay, N = number, OR = odds ratio, SE = standard error.

β, SE, and *P* value estimated using regression analysis adjusted for age, sex, past medical history, cause of trauma, frequency of operation.

OR and 95% CI estimated using logistic regression analysis adjusted for age, sex, past medical history, cause of trauma, frequency of operation.

For the first model, the effect that the ISS subgroup and the involvement of the PS department had on prognosis was assessed. The LOS of the ISS < 25 subgroup was 13.510 days shorter than that of the reference group (Non-PS and ISS ≥ 25, *P* < .001), and the ICU LOS was 7.292 days shorter than that of the reference group (Non-PS and ISS ≥ 25, *P* < .001). Additionally, PS involvement also affected the rate of discharge to home in the same subgroup (OR = 3.243, 95% CI = 1.919–5.480 for the PS group and ISS < 25 subgroup and OR = 1.391, 95% CI = 0.791–2.447 for the non-PS group and ISS < 25 subgroup).

For the second model, the effect that a facial injury on the AIS and the involvement of the PS department had on prognosis was assessed; however, only the rate of discharge to home was correlated with the PS group and facial injury on the AIS (OR = 3.035, 95% CI = 1.985–4.640).

For the final model, the effect that the GCS score and PS involvement had on prognosis was assessed. For the analysis of the total LOS, the group with a GCS score ≥ 13 was correlated with the duration of hospitalization only when the PS department was involved (β = −11.264, *P* = .014 in the PS and GCS ≥ 13 group and β = −4.146, *P* = .368 in the Non-PS and GCS ≥ 13 group). In addition, the synergic effect of the combination of a higher GCS score and PS involvement was found only for the analysis of the rate of discharge to home. The patients with a GCS score ≥ 13 in the PS group were discharged to home 2.20 times more than those in the non-PS and ≥ 13 GCS group (OR = 4.714, 95% CI = 2.203–10.085 vs OR = 2.144, 95% CI = 1.001–4.593). There was no significant difference among the subgroups for any of the 3 models for the disability diagnosis analysis.

## 4. Discussion

According to the 2019 annual report on the Cause of Death Statistics in Korea, 53.1 subjects per 100,000 died due to trauma. Specifically, 21.2% of the working-age population (aged 15–64 years) died due to trauma;^[3]^ therefore, trauma or death in this age group is not only a huge health burden, but also an economic burden. For the above reasons, the treatment of trauma patients has emerged as a social problem, and trauma centers have been established so patients can be treated anywhere in the country within the golden time. Since the establishment of the first trauma center in Korea in 2012, 17 regional trauma centers have been developed. The trauma center aims to benchmark the Level 1 Trauma Center in the United States and the National Health Service Trauma Center in the United Kingdom, advocating holistic and multidisciplinary care for trauma patients, and lowering the preventable trauma death rate (PTDR). The PTDR of countries with advanced medicine, such as the United States, the United Kingdom, and Germany, was about 15%, while it was close to 35% in Korea in 2010.^[4]^ However, in 2017, 5 years after the establishment of the Korea Regional Trauma Center, the PTDR was 19.9%, a significant improvement in a short period of time.^[5]^

As plastic surgeons are involved in both elective and emergency surgery, they figure significantly in treating major trauma patients, who often suffer from fractured facial bones, injuries of bodily soft tissue, and injuries to the hand or neurovascular injuries. Our previous study statistically explored the contribution of plastic surgeons to trauma patient management. The study found that approximately 5% of all emergency room patients were treated by plastic surgeons, while the proportion rose to roughly 15% for trauma patients.^[6]^ Due to their approach to reconstruction and rehabilitation, plastic surgeons are uniquely positioned to play a vital role in a subspecialty for trauma reconstruction. The improvement in outcomes when coordinated care is provided from the resuscitation to rehabilitation stage is exemplified in open lower limb fractures. A combined effort from orthopedic and plastic surgeons for early soft tissue reconstruction in such fractures is established as the gold standard of care in the United Kingdom, as described in the national guidelines set out in February 2016 by the National Institute for Health and Care Excellence. This underscores the importance of clinicians and multidisciplinary health care professionals collaborating for the patient’s journey extending beyond immediate treatment of injury. Plastic surgeons also play a crucial role in such cases, in addition to dealing with nonfatal disease.^[7]^

Trauma severity scoring systems can help to improve communication among medical staff through the quantification of various and complex injuries, as well as preparing guidelines such as prioritizing severe trauma treatment. These scoring systems are also used for assessing risk adjustment, which is a fundamental premise of therapeutic performance research.^[8]^ Historically, many trauma severity scoring systems have been proposed since the 1970s, when trauma center systems began to be developed in the United States. Such systems are usually divided into anatomical, physiological, and combined systems. The ISS, which was created by Baker et al in 1974, has been considered the “gold standard” among the available anatomic injury severity indicators.^[9]^ Additionally, since the GCS was introduced in 1974, it has been widely used to assess patients’ physiological condition, as head and face trauma is a common cause of mortality and morbidity in patients with severe trauma.^[10]^ The Trauma Injury Severity Score and A Severity Characterization of Trauma are examples of combined indicators.

Such indicators focusing on the mortality rate, however, are not able to gauge the effect of trauma care over longer periods of time, complicating the evaluation of the lasting impact of trauma among survivors. While Trauma Quality-of-Life is the only measure of long-term outcomes of trauma that incorporates a biopsychosocial perspective on patient care, it is not applied due to its inconveniently lengthy duration of measurement, lack of composite scores, and uncertain validity. Other more specific yet generalizable methods with more reliability and tested validity for all trauma populations are currently being studied.^[11]^ Since the PS department does not handle patients’ vital signs directly and evaluating the quality of life of trauma patients is more meaningful, we focused on qualitative indicators of prognosis, such as discharge disposition and disability diagnosis. In addition, common quantitative indicators of prognosis, such as LOS and ICU admission rates, were simultaneously evaluated.

Among the qualitative prognoses assessed, only discharge disposition had significant correlations based on the participation of the PS department. Similarly, previous studies have argued that discharge disposition is affected by age, ISS, ICU LOS, and facial injuries.^[12,13]^ The reason for the high rate of discharge to home in the PS group was that while this population usually suffered facial injuries, they could often ambulate. Therefore, it was difficult to conclude whether the patients’ satisfaction with PS was significantly high, as the rate of disability diagnosis was similar in both groups.

One of the limitations of this study was its design as a retrospective study in a single center. A second limitation is that while we were able evaluate trauma patients’ severity indicators with the ISS and GCS, there was limited information regarding the patients’ overall condition. In addition, since the severity evaluation index for Korea has not yet been established, it is expected that more accurate data will be available only when the severity evaluation tool for those who treated in Korea is developed. A third limitation is the variation in the triage of trauma patients depending on the hospital or region. For instance, while our hospital mainly treats patients with facial bone fractures, in England, lower extremity trauma is dominant.^[6,14]^ Nevertheless, a major strength of this study was the analysis of the characteristics and qualitative indicators of prognosis in patients with PS involvement among severe trauma patients, and these differences were recognized through a comparison with controls.

In conclusion, this study was conducted to better understand the effect of PS on the qualitative prognoses of severe trauma patients. We found that higher trauma severity generally affected the prognoses of severe trauma patients, and that PS involvement only significantly contributes to discharge disposition to home.

## Author contributions

**Conceptualization:** Nam Kyu Lim.

**Data curation:** Sungyeon Kim, Jae Hee Yoon.

**Formal analysis:** Nam Kyu Lim, Jae Hee Yoon.

**Investigation:** Nam Kyu Lim, Sungyeon Kim, Jae Hee Yoon, Kyung-Hwa Choi.

**Methodology:** Nam Kyu Lim, Kyung-Hwa Choi.

**Project administration:** Nam Kyu Lim.

**Resources:** Nam Kyu Lim.

**Software:** Kyung-Hwa Choi.

**Supervision:** Nam Kyu Lim, Kyung-Hwa Choi.

**Validation:** Nam Kyu Lim, Kyung-Hwa Choi.

**Visualization:** Nam Kyu Lim, Kyung-Hwa Choi.

**Writing – original draft:** Nam Kyu Lim, Jae Hee Yoon.

**Writing – review & editing:** Nam Kyu Lim.
